# Research Trends and Core Themes in Operating Room Patient Safety: A Scope-Based Keyword Network Analysis (2020–2024)

**DOI:** 10.3390/healthcare13233164

**Published:** 2025-12-03

**Authors:** Ribyeol Woo, Jieun Shin, Nam-Yi Kim

**Affiliations:** 1 College of Nursing, Konyang University, Daejeon 35365, Republic of Korea; ribyeol@kyuh.ac.kr; 2 Department of Biomedical Informatics, College of Medicine, Konyang University, Daejeon 35365, Republic of Korea; jeshin@konyang.ac.kr

**Keywords:** patient safety, operating room, safety culture, scope-based keyword network analysis, teamwork

## Abstract

**Background:** Operating rooms are high-risk environments where ensuring patient safety is essential. Although research on patient safety has increased in recent years, comprehensive analyses of research trends and the core topics specific to operating room safety remain limited. This study aimed to analyze the core keywords and network structures in operating room patient safety, using a scope-based approach to provide suggestions for future research and practice. **Methods**: We conducted a scope-based keyword network analysis of studies on operating room patient safety published between 2020 and 2024. Data were collected from major academic databases, including CINAHL, MEDLINE, and PubMed. Keyword frequency and network centrality measures (degree, closeness, and betweenness) were used to identify major keywords and their interrelationships. **Results**: The analysis revealed ‘patient safety’, ‘operating room’, and ‘nurse’ as the most frequent and central keywords, highlighting their critical role in surgical safety research. Other highly connected terms—‘safety culture’, ‘infection control’, and ‘checklist’ emphasized systematic and organizational safety management. Emerging themes such as ‘leadership’, ‘teamwork’, ‘competency’, and ‘education’ reflected increasing attention to collaboration and professional capability, while ‘artificial intelligence’ and ‘telemedicine’ indicated growing interest in digital innovation. **Conclusions**: Research on patient safety in operating rooms demonstrates a multidimensional structure encompassing patients, healthcare professionals, systems, culture, and education. These findings underscore the need for integrated and multidisciplinary approaches to enhance safety in surgical environments and suggest directions for technology-driven and patient-centered safety models.

## 1. Introduction

Patient safety is a key priority emphasized by the World Health Organization and by healthcare institutions worldwide, particularly regarding operating rooms, which are classified as high-risk environments owing to the increased likelihood of patient safety incidents [1]. Adverse events during surgical procedures can result in fatal outcomes for patients. This necessitates the continuous improvement of systematic patient safety management strategies by medical institutions.

According to patient safety incident reports published by the Korea Institute for Healthcare Accreditation, incidents occurring in operating rooms have shown a continual increase [2]. Indeed, a significant proportion of patient safety events reported both domestically in Korea and internationally originate in surgical settings. Such repeated incidents persist because of the limitations of existing preventive safety systems [3].

Recent studies have proposed various approaches to analyze the factors that contribute to surgical incidents and enhance safety management. Lebni et al. investigated the impact of human and environmental factors on medical errors in operating rooms. They reported that systematic training and environmental improvements were effective in preventing adverse events [4]. Baurasien et al. also suggested that real-time patient monitoring systems utilizing artificial intelligence and big data technologies have a positive impact on improving operating room safety [5]. While these studies provide insights into individual factors and interventions, they still have limitations in establishing integrated strategies that connect findings across studies.

Keyword network analysis based on big data has increasingly become effective method for identifying research trends and key issues across various disciplines [6]. This approach enables the visualization of relationships among key terms and the assessment of centrality, thereby facilitating an integrated understanding of research directions and patterns. Despite active research on operating room patient safety over the past five years, comprehensive analyses that synthesize trends and identify core keywords remain limited [7].

Therefore, this study aimed to conduct a scope-based keyword network analysis of the literature related to patient safety in operating rooms published from 2020 to 2024. By identifying the relationships among key concepts, this study sought to clarify the current landscape of patient safety issues in surgical environments and to suggest future research directions. To achieve these aims, the study addressed the following research questions:

(1) What are the recent research trends related to patient safety in operating rooms?
(2) Which keywords and themes form the core structure of operating room patient safety research?
(3) Based on the keyword network analysis, what implications and future research directions can be identified for improving operating room safety?

Specifically, this study presents visual representations of keyword centrality and co-occurrence strength to map the structure of patient safety research in operating rooms. These results provide insights that can generate new research themes and support researchers and healthcare institutions in establishing effective safety management strategies.

## 2. Materials and Methods

This study aims to systematically examine research trends related to patient safety in operating rooms using network text analysis. The research methodology consisted of four primary stages: literature collection, data preprocessing, keyword extraction, and network construction and analysis. Each stage is described in detail below.

### 2.1. Data Collection

Relevant studies were retrieved from the domestic and international academic databases. For the Korean literature, we used the Research Information Sharing Service (RISS) provided by the Korea Education and Research Information Service. For the international literature, databases including CINAHL, MEDLINE, and PubMed were searched. The search was limited to articles published within the past five years, from 2020 to 2024.

Considering the specificity of operating room patient safety research within nursing and healthcare contexts, these three databases were deemed sufficient to comprehensively capture relevant literature. Therefore, the search scope was maintained, and the methodological approach was clarified as a scope-based keyword network analysis designed to map the structural landscape and relationships among key concepts in operating room patient safety research. This approach aligns with prior studies in nursing and patient safety, such as Kim et al. [8] and Pollock et al. [9].

The literature search was conducted between June and October 2024, and the publication period was limited to studies published from 2020 to 2024. Because the year 2024 was not yet complete at the time of data collection, the number of publications for that year may appear lower than the actual annual total.

To ensure comprehensive coverage, both Korean and English search terms were applied. The main Korean search terms included ‘환자안전 (patient safety)’, ‘수술실 (operating room)’, ‘안전문화 (safety culture)’, ‘감염관리 (infection control)’, and ‘간호 (nursing)’. English search terms included ‘patient safety’, ‘operating room’, ‘safety culture’, ‘infection control’, and ‘nursing’. In addition, combinations such as ‘surgery AND patient safety’, ‘operating room AND safety’, ‘safety AND perioperative’, and ‘medical error’ were tested to capture relevant variations. These terms were selected based on their frequent use in previous nursing and patient safety studies (Kim et al., 2023 [8]; Pollock et al., 2021 [9]) and were verified across databases to ensure consistency.

A total of 491 articles were initially identified. After removing duplicates (*n* = 118), studies with missing or unidentifiable keywords (*n* = 34), articles written in neither Korean nor English (*n* = 23), and those unrelated to the subject (*n* = 249), a final sample of 67 articles (137 keywords) was included in the analysis. Although expanding the search to include additional synonyms (e.g., ‘surgical safety’, ‘perioperative safety’) could slightly alter the number of retrieved records, cross-database validation confirmed that such variations would not materially affect the overall keyword network structure.

### 2.2. Data Preprocessing

Text data were extracted from the titles, abstracts, and keywords of selected articles. Non-informative words such as stop words, particles, and conjunctions were removed. A cleaning process was then applied to correct typographical errors and to unify similar or duplicate keywords. For example, terms such as ‘Abdomen/surgery’ and ‘Digestive System Surgical Procedures’ were combined into ‘Abdomen surgery’. Similarly, ‘Adverse Health Care Event-Prevention and Control’, ‘Medical errors’, and ‘Medical mistake’ were unified as ‘Adverse event’. The terms ‘Clinical Competence’, ‘Competencies’, and ‘Competency’ were consolidated into ‘Competency’. A complete list of original and merged keywords used in the preprocessing step is provided in the Supplementary Materials, Table S1, to ensure the transparency and reproducibility of the data-cleaning process.

### 2.3. Keyword Extraction and Frequency Analysis

Major keywords were extracted from each article using both author-assigned keywords and text-mined terms from the titles and abstracts to identify trends in the literature. Text-based keyword extraction was conducted using the R “tm” package (Text Mining), and frequency analysis was performed in R version 4.3.1. During preprocessing, non-informative words such as stop words, conjunctions, and prepositions were removed, and synonymous terms were unified to ensure consistency (e.g., “adverse event,” “medical error,” and “patient harm”). After automatic extraction, two independent researchers reviewed the list of keywords and manually refined it to merge conceptually equivalent terms. Discrepancies between researchers were resolved through discussion, and inter-rater reliability was confirmed with Cohen’s κ = 0.86, indicating a high level of agreement. Korean and English keywords were collected separately and later merged when equivalent terms were identified. The most frequently observed keywords included patient safety, operating room, infection control, risk management, and surgical complications.

### 2.4. Keyword Network Construction

A keyword co-occurrence network was constructed by linking keywords that appeared in the same article. The strength of each link was determined based on the frequency of co-occurrence. A network graph was then built with keywords represented as nodes and co-occurrence relationships represented as edges to visualize the interrelationships among the research topics.

Three centrality metrics were used to analyze the network: degree, closeness, and betweenness centralities [10]. The number of nodes represented the total number of unique keywords, while the number of edges indicated the number of keyword-to-keyword connections.

#### 2.4.1. Degree Centrality

Degree centrality quantifies the number of direct connections that a keyword has with other keywords in a network. For node *i*, degree centrality is defined as:

$$\mathrm{Degree}\;\mathrm{Centrality}\;C_{D}\left(i\right)=\frac{deg(i)}{n-1}$$

where deg(*i*) is the number of edges connected to node *i*, and *n* is the total number of nodes.

#### 2.4.2. Closeness Centrality

Closeness centrality measures the closeness of a keyword to all other keywords in a network based on the shortest path distance. For node *i*, it is defined as:

$$\mathrm{Closeness}\;\mathrm{Centrality}\;C_{C}\left(i\right)=\frac{n-1}{\sum_{j\neq i}d(i,j)}$$

where d(i,j) is the shortest path between nodes *i* and *j*. A higher closeness centrality implies that the node is located closer to the center of the network and is more easily accessible from other nodes.

#### 2.4.3. Betweenness Centrality

Betweenness centrality reflects the extent to which a keyword lies on the shortest path between other pairs of keywords. For node *i*, it is calculated as:

$$\mathrm{Betweenness}\;\mathrm{Centrality}\;C_{B}\left(i\right)=\sum_{s\neq i\neq t}\frac{\sigma_{st}(i)}{\sigma_{st}}$$

where σ_*st*_ is the number of shortest paths from nodes *s* to *t*, and σ_*st*_(i) is the number of those paths that pass through node *i*.

### 2.5. Network Analysis and Visualization

Using the constructed keyword network, we analyzed research trends and interrelationships among the key concepts. Major themes and clusters were identified, and their relationships were interpreted. A visual network graph was created to intuitively display the research trends and key issues.

We also analyzed the annual distribution of publications to observe research trends over time. In addition, the frequency of keywords was examined to identify key terms. The SNA package in R (version 4.3.1) was used to conduct network text analysis and visualize keyword associations. Moreover, ego network analysis was performed to further explore the local structure surrounding core keywords by examining their direct connections and relational patterns with neighboring terms.

## 3. Results

### 3.1. Research Trends and Keyword Analysis

**Figure 1:**
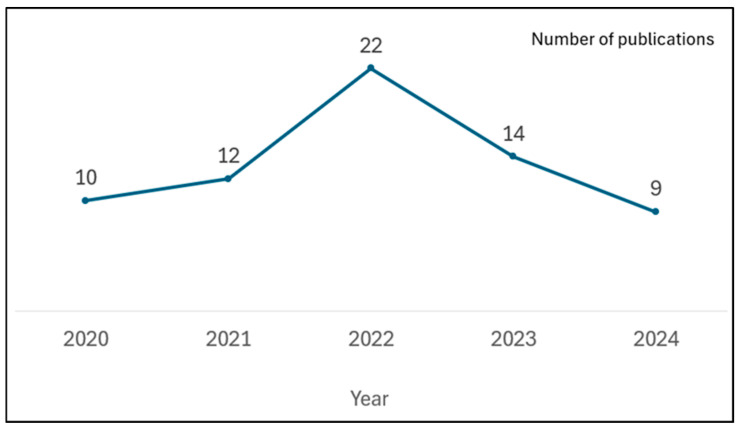
Annual number of publications related to patient safety in operating rooms, 2020–2024 (note: data for 2024 were collected in October 2024 and may not represent the full annual total).

Over the past five years (2020–2024), the number of publications related to patient safety in operating rooms has fluctuated. The annual number of publications increased from 10 in 2020 to a peak of 22 in 2022, followed by a decrease to 14 in 2023 and 9 in 2024, as shown in Figure 1.

The keyword network analysis revealed that over the past five years (2020–2024), the most central keyword in the literature related to patient safety in operating rooms was ‘patient safety’. This keyword was recorded to have the highest values across all centrality measures, including frequency (*n* = 41), degree centrality (145), closeness centrality (0.7637), and betweenness centrality (6532.7), strongly indicating its role as the core topic in this field.

The keyword ‘operating room’ (*n* = 25) ranked second in all centrality indices, suggesting that the operating room itself remains a primary focus directly associated with patient safety. Other keywords with high centrality values included ‘humans’, ‘perioperative care’, ‘nurse’, and ‘operating room nurses’, highlighting the importance of healthcare personnel and perioperative management in surgical safety research. Notably, ‘perioperative care’ ranked third in closeness centrality (0.6803), indicating that integrated care throughout the perioperative period is closely connected to other concepts and serves as a key thematic area that encompasses nursing interventions and leadership.

Keywords such as ‘checklist’, ‘safety management’, ‘surgical site infection’, and ‘health care worker’ also demonstrated strong centrality, reflecting the relevance of standardized safety protocols, infection prevention, and nursing workforce culture in promoting patient safety.

In terms of betweenness centrality, ‘patient safety’, ‘operating room’, ‘humans’, and ‘perioperative care’ function as key mediators in the network, bridging various concepts. This suggests that these keywords not only represent central research themes but also facilitate connections between subtopics, thereby playing a pivotal role in shaping the overall direction of research in this domain (Table 1).

### 3.2. Ego Network Analysis

In the ego network analysis, we examined the relationships between the core keywords (egos) ‘patient safety’, ‘operating room’, ‘humans’, and ‘perioperative care’, and their directly connected keywords (alters). Forty-one studies that included the term ‘patient safety’, 25 that included ‘operating room’, and 16 that included ‘perioperative care’ were analyzed.

### 3.3. Patient Safety

The keyword ‘patient safety’ was directly connected to several highly central keywords, including ‘checklist’, ‘surgery’, ‘leadership’, ‘operating room nurses’, ‘safety management’, and ‘Delphi method’. All of these terms exhibited high degrees and high closeness centrality values. Notably, ‘checklist’ (degree = 15, closeness = 0.6104) reflects sustained academic attention to standardized safety verification procedures. This can also be interpreted in relation to the implementation of the World Health Organization’s Surgical Safety Checklist.

‘Leadership’ (degree = 12, closeness = 0.5958) and ‘operating room nurses’ (degree = 12, betweenness = 7.0) highlight the strong association between the leadership and expertise of surgical staff—particularly nurses—and patient safety outcomes. Other connected terms, such as ‘safety management’, ‘Delphi method’, ‘attitude of health personnel’, and ‘teamwork’, represent concepts that expand into organizational culture, team-based collaboration, and expert consensus-based intervention strategies, indicating practical and educational implications for patient safety practices.

In particular, the ‘Delphi method’ demonstrated a high centrality score (e.g., closeness = 0.5958), indicating that expert panel consensus methods are actively utilized in the development of measurement tools and intervention components related to operating room patient safety (Table 2).

### 3.4. Operating Room

In the ego network analysis, the keyword ‘operating room’ was recorded to have the highest centrality scores within the network. It appeared 25 times across the dataset, with a degree centrality of 94, a closeness centrality of 0.9698, and a betweenness centrality of 1625.7, each representing the highest values among all keywords. These metrics indicate that ‘operating room’ functions as a core concept in research on patient safety in surgical settings and plays a pivotal role as a central hub in the network of related keywords.

Keywords directly connected to ‘operating room’ included ‘patient safety’, ‘humans’, ‘health personnel’, ‘organizational culture’, ‘nurse’, ‘safety management’, ‘attitude of health personnel’, ‘distractions’, ‘COVID-19’, and ‘health care worker’. These terms demonstrated a relatively high degree of closeness centrality, suggesting that they maintained strong and meaningful relationships with the core keywords in the network. In particular, ‘patient safety’ (degree = 63, closeness = 0.8755, betweenness = 727.0) showed the second-highest centrality values, reflecting its strong association with safety issues in the operating room environment. ‘Humans’ (degree = 25, closeness = 0.7255, betweenness = 229.8) also occupied a significant position, indicating a focus on human factors within surgical safety research. Additionally, ‘health personnel’ (degree = 14, closeness = 0.6958, betweenness = 98.5) and ‘nurse’ (degree = 12, closeness = 0.6018, betweenness = 97.0) were identified as key actors in practical patient safety efforts.

Other important keywords included ‘safety management’ (degree = 13, closeness = 0.4894, betweenness = 84.0), ‘attitude of health personnel’ (degree = 11, closeness = 0.5940, betweenness = 97.0), and ‘distractions’ (degree = 9, closeness = 0.4563, betweenness = 119.0), which reflect themes related to safety systems, behavioral factors, and environmental interruptions in the operating room. The emergence of the keyword ‘COVID-19’ (degree = 9, closeness = 0.4760, betweenness = 53.3) also reflected the influence of the recent pandemic on surgical environments and patient safety practices (Table 3).

### 3.5. Humans

In the ego network analysis, the keyword ‘humans’ was recorded to have the highest closeness centrality (1.0907) among all keywords, while also ranking high in degree centrality (69) and betweenness centrality (605.5). With a frequency of 16, this result indicates that research on operating room patient safety frequently involves human participants and healthcare personnel, reflecting the broad relevance of human-related aspects within this field. Although this may suggest potential connections to human-factors or ergonomics research, the term ‘humans’ is inherently broad and should be interpreted cautiously rather than as direct quantitative evidence of a specific disciplinary theme.

‘Humans’ was directly connected to several key keywords, including ‘patient safety’, ‘operating room’, ‘perioperative care’, ‘postoperative complications’, ‘leadership’, ‘safety management’, ‘health personnel’, ‘organizational culture’, ‘physicians’, and ‘patients’. Many of these terms also function as central keywords in the network, based on their high degree and closeness centrality. Notably, ‘patient safety’ (degree = 49, closeness = 0.9890, betweenness = 293.5) was strongly connected to ‘humans’, indicating a close association with safe nursing practices. ‘Operating room’ (degree = 25, closeness = 0.7997, betweenness = 123.0) reflected topics related to human factors in the surgical environment, while ‘perioperative care’ (degree = 20, closeness = 0.8855, betweenness = 88.5) highlighted links between pre- and postoperative care and human-centered nursing. Additional terms such as ‘leadership’ (degree = 12, closeness = 0.6186), ‘safety management’ (degree = 12, closeness = 0.5175), ‘health personnel’ (degree = 10, closeness = 0.5703), and ‘organizational culture’ (degree = 13, closeness = 0.6804) further illustrated how organizational and cultural dimensions interact with human capabilities in the context of patient safety.

Other keywords directly linked to ‘humans’ included ‘physicians’, ‘patients’, ‘surveys and questionnaires’, ‘telemedicine’, ‘cognition’, and ‘arrhythmias’. Some of these keywords exhibited a betweenness centrality of 0.0, suggesting that they functioned more as peripheral nodes in the network than as intermediaries (Table 4).

### 3.6. Perioperative Care

In the ego network analysis, the keyword ‘perioperative care’ appeared 16 times, with a degree centrality of 58, closeness centrality of 0.9632, and betweenness centrality of 403.8. These metrics suggests that the domain of integrated care across the pre-, intra-, and postoperative phases is a key concept in research on patient safety in operating rooms.

The major keywords directly connected to ‘perioperative care’ included ‘patient safety’, ‘humans’, ‘leadership’, ‘Delphi method’, ‘operating room nurses’, ‘education’, ‘quality of nursing care’, ‘anesthesia’, and ‘consensus’. Among these, ‘patient safety’ (degree = 49, closeness = 0.9542, betweenness = 234.3) was one of the most strongly linked terms, suggesting that the concept of safety is tightly embedded within perioperative nursing practices. ‘Humans’ (degree = 20, closeness = 0.8216, betweenness = 117.8) reflected the connection to human factors, while ‘leadership’ (degree = 12, closeness = 0.5583, betweenness = 167.5) indicated that nursing leadership is a prominent topic in perioperative nursing research. The ‘Delphi method’ (degree = 9, closeness = 0.5563, betweenness = 0.5) was frequently used in the development of perioperative safety indicators and evaluation tools. Other keywords included ‘operating room nurses’ (degree = 7, closeness = 0.3967), ‘education’ (degree = 5, closeness = 0.3967), ‘anesthesia’ (degree = 7, closeness = 0.3935), and ‘consensus’ (degree = 9, closeness = 0.3923).

Additional connected keywords were ‘health behavior’, ‘competency’, ‘patients’, ‘speaking up’, ‘communication’, ‘delirium’, ‘hydrocortisone’, ‘Guatemala’, ‘questionnaire’, and ‘psychometric properties’. Most of these keywords exhibited a betweenness centrality of 0.0, indicating that although they were directly connected to ‘perioperative care’, they did not function as intermediaries in the overall flow of information within the network (Table 5).

### 3.7. Thematic Evolution by Publication Period

**Figure 2:**
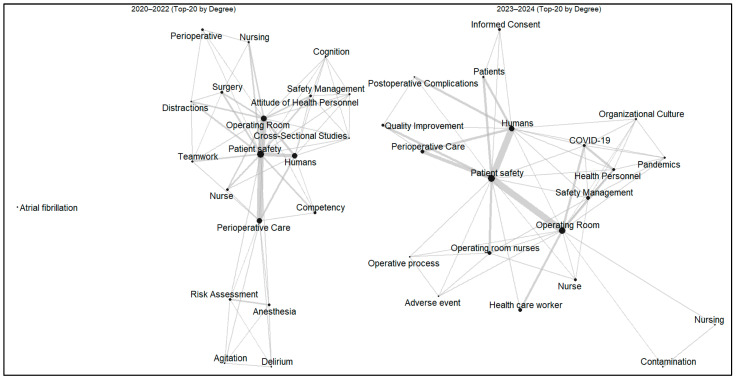
Keyword co-occurrence networks of perioperative patient safety research by publication period (2020–2022 vs. 2023–2024). (Note: Node size represents degree centrality, and edge thickness indicates co-occurrence strength).

To explore temporal changes in research themes, the literature was divided into two periods (2020–2022 and 2023–2024), and keyword co-occurrence networks were constructed for each period (Figure 2). In both networks, patient safety, operating room, and perioperative care consistently exhibited the highest degree centrality, indicating their central position within the research field (Tables S2 and S3).

During 2020–2022, studies predominantly focused on human and behavioral factors influencing perioperative safety. Keywords such as competency, attitude of health personnel, and teamwork were strongly linked to patient safety and operating room, reflecting early research attention to professional capability and intra-team communication within the surgical context.

In contrast, the 2023–2024 network revealed a more diversified and system-oriented structure. Alongside the core cluster of patient safety and operating room, new central keywords such as safety management, organizational culture, COVID-19, and quality improvement emerged. This shift suggests a thematic evolution from individual-level competency and teamwork toward organizational strategies and quality systems that enhance safety in perioperative environments.

## 4. Discussion

This study conducted a keyword network analysis of the literature on operating room patient safety published over the past five years (2020–2024) to identify research trends and structural relationships among the core concepts. The analysis revealed that patient safety had the highest values across all the centrality measures, confirming its role as a central concept in this research domain. This finding underscores the prioritization of patient safety in healthcare environments and aligns with the objectives outlined in the World Health Organization’s Global Patient Safety Action Plan 2021–2030.

### 4.1. Interpretation of Core Keywords and Centrality Analysis

The prominence of the keywords ‘patient safety’, ‘operating room’, ‘humans’, and ‘perioperative care’ in both the frequency and centrality measures suggests that patient safety in operating rooms is being studied as an integrated concept that extends beyond isolated incident prevention to encompass the entire perioperative process and human factors. In particular, the high closeness centrality of ‘perioperative care’ highlights the increasing recognition of continuous preoperative and postoperative nursing care as core elements of patient safety. This aligns with the findings of Gabriel et al. [11], who emphasized the impact of uninterrupted care from presurgical preparation to postoperative management on patient outcomes.

However, the interpretation of the keyword ‘humans’ requires careful consideration. The term is inherently broad and may not necessarily refer to ‘human factors and ergonomics’ as a specific discipline, but rather to studies involving human participants, healthcare personnel, and clinical practices more generally. Thus, its high centrality likely reflects the broad inclusion of human-related dimensions in operating room patient safety research, rather than serving as direct quantitative evidence of a particular subfield. This highlights the importance of terminological precision in bibliometric network analysis, as different sub-domains may form unique conceptual clusters [12]. Future studies should incorporate content analysis to clarify how ‘humans’ is conceptually represented across different research contexts.

The strong positioning of nurse-related keywords such as ‘nurse’ and ‘operating room nurses’ underscores the central role of nursing professionals in ensuring surgical safety. According to Steelman et al. [13], the expertise, experience, and communication competence of operating room nurses are directly associated with patient safety outcomes. This is further supported by Wu et al. [14], who described operating room nurses as frontline actors in patient safety management and emphasized the importance of strengthening their competencies to foster a robust safety culture. Notably, the high betweenness centrality of ‘operating room nurses’ indicates their critical role in bridging various safety-related topics.

The centrality of keywords such as ‘safety management’, ‘checklist’, and ‘safety culture’ further reflects a strong academic focus on systematic safety management and the development of organizational safety culture. This corresponds with Haynes et al.’s [15] influential work on the effectiveness of the Surgical Safety Checklist, reinforcing the view that safety should be embedded not only in procedures but also in institutional culture. The presence of terms such as ‘quality improvement’ and ‘organizational culture’ suggests an ongoing shift toward system-level approaches for improving patient safety. This is consistent with the findings of Odell et al. [16], who reported that fostering a strong safety culture and organizational framework contributed significantly to the reduction in adverse events.

### 4.2. Digital Technology and Innovation in Patient Safety

A noteworthy finding from the network analysis was the appearance of keywords such as ‘artificial intelligence’, ‘telemedicine’, and ‘technology’. This indicates that digital innovation is emerging as a new research theme within the field of operating room patient safety. Studies by Ferrara et al. [17] and Bellini et al. [18] have reported that artificial intelligence-based decision support systems are effective in predicting and responding to intraoperative risks. Safety management using real-time monitoring and dashboard systems has become a new paradigm.

In recent clinical settings, various technologies such as virtual-reality-based surgical simulations, robot-assisted surgery, and wearable devices for monitoring the fatigue of healthcare providers have been introduced. However, the relatively low centrality scores of these keywords in the network analysis suggest that research in this area is still in its early stages. Grygorian et al. [19] also emphasized the need for additional empirical studies on the impact of digital technologies on patient safety.

### 4.3. Infection Control and the Impact of COVID-19

The prominence of infection-related keywords such as ‘surgical site infection’ and ‘staphylococcal infections’ indicates that surgical site infections remain a critical issue in patient safety. Notably, the keyword ‘COVID-19’ exhibited a high betweenness centrality, suggesting that the pandemic had a substantial impact on operating room environments and clinical protocols. In their 2024 study, Ferreira et al. [20] reported significant changes in infection control protocols and shifts in patient safety paradigms following the onset of COVID-19 in surgical settings.

The pandemic affected not only infection control within the operating room, but also broader aspects such as surgical scheduling, resource allocation, and healthcare worker safety. According to Wong et al. [21], surgery delays and cancellations caused by COVID-19 have indirect negative effects on patient safety, posing new challenges for surgical operations in the post-pandemic era. The emergence of the keyword ‘pandemics’ further underscores the need for strategic planning in operating room safety in preparation for future infectious disease outbreaks.

### 4.4. Patient-Centered Approaches and Interdisciplinary Collaboration

The high centrality of keywords such as ‘leadership’, ‘teamwork’, and ‘competency’ reflects the importance of multidisciplinary approaches and team-based collaboration in ensuring patient safety. This finding aligns with the team-based strategies for reducing medical errors proposed by Makary and Daniel [22]. In particular, the high betweenness centrality of ‘competency’ (279.0) highlights that the development of the competencies of healthcare professionals serves as a key linking concept across various patient safety themes.

The emergence of ‘distractions’ (betweenness centrality = 189.0) suggests that sources of attentional disruption in the operating room are increasingly being recognized as threats to patient safety. This is consistent with the findings of Wheelock et al. [23], who reported that distractions in the surgical setting negatively affect the performance of healthcare providers. In this study, ‘distractions’ was strongly associated with both ‘patient safety’ and ‘operating room’, reinforcing the notion that distraction management is a critical component of safety in the surgical environment. Interestingly, the presence of keywords such as ‘informed consent’ and ‘person-centered care’ in the network suggests that patient engagement and empowerment are emerging as important elements of surgical safety. According to Haugen et al. [24], involving patients and caregivers as safety partners contributes to improved surgical outcomes. This trend indicates a paradigm shift from traditional provider-centered safety frameworks to more collaborative, patient-centered approaches.

### 4.5. The Importance of Education and Simulation

The presence of keywords such as ‘education’ and ‘simulation’ in the network highlights the growing emphasis on education in the context of operating room patient safety. According to Fong et al. [25], simulation-based education improves crisis response capabilities and enhances teamwork among surgical teams. Notably, the connection between ‘education’ and ‘organizational culture’ observed in the network suggests that educational approaches at the organizational level play a vital role in fostering a culture of safety. Beyond traditional lecture-based instruction, a range of educational strategies, including practice-based learning, team training, and crisis management simulations, are increasingly being implemented in clinical settings. Pons et al. [26] reported that repeated simulation training enhanced the nontechnical skills of operating room staff, thereby contributing to patient safety. The identification of strong connections between education-related keywords in the current network analysis further indicates that educational strategies have become an essential focus within the field of patient safety research.

### 4.6. Thematic Evolution in Perioperative Patient Safety Research

The temporal analysis revealed a distinct thematic shift in perioperative patient safety research. From 2020–2022, studies primarily focused on competency, attitude of health personnel, and teamwork, reflecting micro-level attention to human performance and communication within surgical teams. In contrast, the 2023–2024 network demonstrated an expansion toward safety management, organizational culture, COVID-19, and quality improvement, signifying a system-level orientation toward institutional safety and resilience.

This evolution suggests that the research field has progressed from identifying individual and procedural risks to developing organizational frameworks that sustain safety culture and quality assurance. Similar transitions have been reported in other healthcare domains where safety is increasingly viewed as an institutional responsibility rather than an individual behavior [27,28].

Overall, the growing emphasis on organizational culture and quality improvement implies a maturation of perioperative safety research toward sustainable and systemic approaches. Future studies should integrate multidisciplinary strategies and evaluate how organizational and policy-level interventions influence patient outcomes safety.

### 4.7. Implications of the Study

This study provides an integrative understanding of recent trends in operating room patient safety research. The findings demonstrate that safety in surgical environments is shaped by the interaction of system-level structures, human factors, and technological innovation. Taken together, several broader implications can be drawn:

- For healthcare practice: Safety management should move beyond compliance-based protocols toward continuous learning systems that integrate teamwork, leadership, and technology into daily surgical practice.
- For education and training: Simulation- and competency-based education should be institutionalized in both academic and clinical settings to cultivate sustainable safety competencies among healthcare professionals.
- For policy and system design: National and institutional patient safety policies should support digital transformation and interprofessional collaboration as strategic components of safety governance.
- For future research: Longitudinal and mixed-methods approaches are needed to examine how digital innovation, team culture, and organizational resilience jointly influence patient outcomes.

In summary, this study underscores the importance of developing integrated, adaptive, and human-centered safety frameworks that align technological progress with the core values of patient care.

### 4.8. Limitations and Directions for Future Research

This study has several limitations. As a keyword-based analysis, it does not fully capture the qualitative aspects of the included studies. In addition, the scope of the literature was limited to articles written in English or Korean, which introduced linguistic constraints. It is suggested that future research incorporate content analysis alongside keyword analysis to provide more in-depth insights and that the scope be expanded to other languages.

Furthermore, because the literature search was completed in October 2024, the number of publications identified for that year may not represent the full annual total. Therefore, the observed decline in 2024 publication counts (Figure 1) should be interpreted with caution, as it reflects the incomplete nature of that year’s data rather than a true decrease in research activity. Additionally, the findings and conclusions of this study should be regarded as exploratory and provisional due to the limited number of analyzed studies (*n* = 67) and the restricted database coverage. The results provide an initial mapping of the current research landscape on operating room patient safety, and further research expanding the dataset and refining the methodology is needed to validate and strengthen these findings.

While this study does not directly test or implement specific interventions to improve operating room safety, it provides a comprehensive overview of existing research domains and gaps. The exploratory evidence presented here may serve as a foundation for risk management teams and researchers to identify underexplored areas, such as organizational culture, teamwork, and system-level safety management. Future studies should aim to translate these bibliometric findings into practical frameworks and intervention models that can enhance perioperative patient safety in real-world settings.

## 5. Conclusions

This study comprehensively analyzed research trends and thematic structures related to operating room patient safety from 2020 to 2024 using a scope-based keyword network approach. The results revealed that patient safety, operating room, and perioperative care consistently occupied central positions in the network, highlighting their fundamental role in this domain. Over time, the research focus evolved from micro-level issues—such as competency, teamwork, and attitude of health personnel—to system- and organization-level themes emphasizing safety management, organizational culture, and quality improvement. This shift reflects a growing understanding of patient safety as an institutional and systemic responsibility rather than an individual task.

The integration of digital and educational dimensions—represented by emerging keywords such as artificial intelligence, telemedicine, leadership, education, and simulation—suggests that technological innovation and professional training are increasingly recognized as key enablers of safety improvement. Furthermore, the influence of *COVID-19* and infection-related terms underscores the importance of adaptive safety management and preparedness for global health crises. Together, these findings indicate that operating room safety research has progressed toward developing multidimensional frameworks that combine human, organizational, and technological elements to strengthen resilience in surgical environments.

Although limited by the number of analyzed publications (*n* = 67) and database coverage, this study provides an initial evidence-based mapping of how operating room patient safety research is evolving in response to digital transformation and changing healthcare systems. Future studies should employ longitudinal and mixed-method designs to examine how leadership, teamwork, and organizational culture interact with technological innovations to enhance patient safety and promote sustainable improvement across perioperative care systems.

## Figures and Tables

**Table 1:** Top 20 keywords by frequency and centrality measures.

Rank	Keyword	*n*	Keyword	Degree	Keyword	Closeness	Keyword	Betweenness
1	Patient safety	41	Patient safety	145	Patient safety	0.7637	Patient safety	6532.7
2	Operating room	25	Operating room	94	Operating room	0.6843	Operating room	3308.5
3	Humans	16	Humans	69	Perioperative care	0.6803	Humans	1610.7
4	Perioperative care	16	Perioperative care	58	Humans	0.6769	Perioperative care	1316.3
5	Nurse	5	Surgery	20	Health personnel	0.4998	Surgery	524.2
6	Operating room nurses	5	Nurse	19	Quality improvement	0.4717	Health care worker	464.7
7	Postoperative complications	5	Safety management	19	Surgery	0.4693	Operating room nurses	461.0
8	Safety management	5	Operating room nurses	17	Checklist	0.4670	Safety management	364.8
9	Surgery	5	Surgical site infection	17	Organizational culture	0.4642	Competency	279.0
10	Checklist	4	Health care worker	16	Delphi method	0.4601	Safety culture	272.0
11	Health care worker	4	Postoperative complications	16	Leadership	0.4587	Informed consent	202.0
12	Nursing	4	Nursing	15	Nurse	0.4431	Nurse	192.5
13	Quality improvement	4	Patients	15	Surgical patient safety	0.4384	Distractions	189.0
14	Safety culture	4	Health personnel	14	Physicians	0.4378	Anesthesia	179.0
15	Staphylococcal infections	4	Competency	13	Safety culture	0.4377	Teamwork	179.0
16	Adverse event	3	Organizational culture	13	Health care worker	0.3667	Nursing	173.7
17	Competency	3	Leadership	12	Safety management	0.3603	Surgical site infection	98.0
18	Health personnel	3	Quality improvement	12	Postoperative complications	0.3603	COVID-19	97.3
19	Leadership	3	Staphylococcal infections	12	Education	0.3599	Operative process	94.0
20	Patients	3	Attitude of health personnel	11	Nursing	0.3575	Checklist	93.5

**Table 2:** Top 20 keywords associated with ‘patient safety’ by frequency and centrality measures.

Rank	Keyword	*n*	Keyword	Degree	Keyword	Closeness	Keyword	Betweenness
1	Patient safety	41	Patient safety	145	Patient safety	1.0525	Patient safety	3222.3
2	Operating room	17	Operating room	63	Perioperative care	0.9173	Operating room	842.8
3	Perioperative care	14	Humans	49	Humans	0.9105	Humans	450.0
4	Humans	11	Perioperative care	49	Operating room	0.8720	Perioperative care	346.8
5	Checklist	4	Surgery	15	Checklist	0.6104	Safety management	233.0
6	Surgery	4	Leadership	12	Surgery	0.6066	Surgery	128.0
7	Leadership	3	Operating room nurses	12	Quality improvement	0.5987	Safety culture	123.0
8	Nurse	3	Attitude of health personnel	11	Delphi method	0.5958	Teamwork	123.0
9	Operating room nurses	3	Nurse	11	Leadership	0.5958	Distractions	119.0
10	Quality improvement	3	Safety management	11	Physicians	0.5642	Nursing	62.0
11	Teamwork	3	Teamwork	11	Health personnel	0.5477	Psychometric properties	62.0
12	Attitude of health personnel	2	Checklist	10	Safety management	0.4584	Checklist	57.5
13	Black box	2	Competency	10	Telemedicine	0.4532	Operating room nurses	7.0
14	Communication	2	Delphi method	9	Organizational culture	0.4413	Attitude of health personnel	3.0
15	Competency	2	Distractions	9	Nurse	0.4413	Quality improvement	2.5
16	Delphi method	2	Physicians	9	Students, medical	0.4397	Patients	1.0
17	Distractions	2	Quality improvement	9	Operating room nurses	0.4377	Adenoma	0.0
18	Health personnel	2	Health personnel	8	Industrial safety	0.4370	Adult	0.0
19	Hospital	2	Hospital	8	Planned behavior theory	0.4370	Adverse event	0.0
20	Nursing	2	Patients	8	Patient readmission	0.4352	Anesthesia	0.0

**Table 3:** Top 20 keywords associated with ‘operating room’ by frequency and centrality measures.

Rank	Keyword	*n*	Keyword	Degree	Keyword	Closeness	Keyword	Betweenness
1	Operating Room	25	Operating Room	94	Operating Room	0.9698	Operating Room	1625.7
2	Patient safety	17	Patient safety	63	Patient safety	0.8755	Patient safety	727.0
3	Humans	5	Humans	25	Humans	0.7255	Safety Management	229.8
4	Health Personnel	3	Health Personnel	14	Health Personnel	0.6958	Distractions	98.5
5	Nurse	3	Organizational Culture	13	Organizational Culture	0.6399	Attitude of Health Personnel	97.0
6	Nursing	3	Safety Management	13	Nurse	0.6018	Nurse	97.0
7	Safety culture	3	Surgery	12	Surgical patient safety	0.6000	Safety culture	89.0
8	Surgery	3	Attitude of Health Personnel	11	Safety culture	0.5940	Humans	85.5
9	Attitude of Health Personnel	2	Nursing	11	Safety Management	0.4894	Surgery	84.0
10	COVID-19	2	COVID-19	10	Surgical site infection	0.4760	COVID-19	53.3
11	Distractions	2	Nurse	10	Health care worker	0.4633	Nursing	41.0
12	Health care worker	2	Distractions	9	Education	0.4578	Organizational Culture	4.2
13	Hospital	2	Hospital	8	Simulation	0.4578	Adverse event	0.0
14	Organizational Culture	2	Safety culture	8	Nursing	0.4572	Animals	0.0
15	Safety Management	2	Cognition	7	Distractions	0.4563	Artificial intelligence	0.0
16	Surgical patient safety	2	Cross-Sectional Studies	7	Surgery	0.4527	Black box	0.0
17	Teamwork	2	Health care worker	7	Adverse event	0.4512	Checklist	0.0
18	Adverse event	1	Pandemics	6	Physicians	0.4475	Co-creation	0.0
19	Animals	1	Surgical patient safety	6	Retained surgical items	0.4457	Cognition	0.0
20	Artificial intelligence	1	Teamwork	6	Technology	0.4427	Competency	0.0

**Table 4:** Top 20 keywords associated with ‘humans’ by frequency and centrality measures.

Rank	Keyword	*n*	Keyword	Degree	Keyword	Closeness	Keyword	Betweenness
1	Humans	16	Humans	69	Humans	1.0907	Humans	605.5
2	Patient safety	11	Patient safety	49	Patient safety	0.9890	Patient safety	293.5
3	Operating Room	5	Operating Room	25	Perioperative Care	0.8855	Operating Room	123.0
4	Perioperative Care	5	Perioperative Care	20	Operating Room	0.7997	Safety Management	88.5
5	Postoperative Complications	4	Organizational Culture	13	Organizational Culture	0.6804	Perioperative Care	7.5
6	Health Personnel	2	Safety Management	13	Physicians	0.6449	Organizational Culture	1.5
7	Leadership	2	Postoperative Complications	12	Leadership	0.6168	Patients	1.0
8	Organizational Culture	2	Health Personnel	10	Health Personnel	0.5703	Abdomen surgery	0.0
9	Patients	2	Leadership	9	Safety Management	0.5175	Adult	0.0
10	Physicians	2	Physicians	9	Telemedicine	0.5127	Arrhythmias	0.0
11	Safety Management	2	Patients	8	Arrhythmias	0.5111	Attitude of Health Personnel	0.0
12	Surveys and Questionnaires	2	Surveys and Questionnaires	8	Quality Improvement	0.4912	Austria	0.0
13	Abdomen surgery	1	Attitude of Health Personnel	7	Students, Medical	0.4762	COVID-19	0.0
14	Adult	1	Cognition	7	Patient Readmission	0.4680	Cognition	0.0
15	Arrhythmias	1	Cross-Sectional Studies	7	Nurse	0.4625	Competency	0.0
16	Attitude of Health Personnel	1	Arrhythmias	6	Pandemics	0.4547	Consensus	0.0
17	Austria	1	COVID-19	6	Consensus	0.4467	Cross-Sectional Studies	0.0
18	COVID-19	1	Defibrillators, Implantable	6	Delphi method	0.4467	Defibrillators, Implantable	0.0
19	Cognition	1	Female	6	Informed Consent	0.4434	Delivery of Health Care	0.0
20	Competency	1	Heart Arrest	6	Adult	0.4413	Delphi method	0.0

**Table 5:** Top 20 keywords associated with ‘perioperative care’ by frequency and centrality measures.

Rank	Keyword	*n*	Keyword	Degree	Keyword	Closeness	Keyword	Betweenness
1	Perioperative Care	16	Perioperative Care	58	Perioperative Care	0.9632	Perioperative Care	403.8
2	Patient safety	14	Patient safety	49	Patient safety	0.9542	Patient safety	234.3
3	Humans	5	Humans	20	Humans	0.8216	Leadership	167.5
4	Leadership	3	Leadership	12	Leadership	0.5583	Humans	117.8
5	Delphi method	2	Delphi method	9	Delphi method	0.5563	Delphi method	0.5
6	Adenoma	1	Agitation	7	Quality of Nursing Care	0.4088	Adenoma	0.0
7	Adult	1	Anesthesia	7	Education	0.3967	Adult	0.0
8	Agitation	1	Delirium	7	Operating room nurses	0.3967	Agitation	0.0
9	Anesthesia	1	Health Behavior	7	Speaking up	0.3967	Anesthesia	0.0
10	Austria	1	Patients	7	Agitation	0.3935	Austria	0.0
11	Communication	1	Risk Assessment	7	Anesthesia	0.3935	Communication	0.0
12	Competency	1	Wounds and Injuries	7	Consensus	0.3923	Competency	0.0
13	Consensus	1	Adult	5	Adult	0.3881	Consensus	0.0
14	Delirium	1	Competency	5	Patient positioning	0.3869	Delirium	0.0
15	Education	1	Education	5	Austria	0.3836	Education	0.0
16	Guatemala	1	Nurse	5	Communication	0.3836	Guatemala	0.0
17	Health Behavior	1	Psychometric properties	5	Guatemala	0.3836	Health Behavior	0.0
18	Hydrocortisone	1	Questionnaire	5	Nurse	0.3795	Hydrocortisone	0.0
19	Hypothalamic Pituitary Adrenal Axis	1	Speaking up	5	Competency	0.3760	Hypothalamic Pituitary Adrenal Axis	0.0
20	Illicit substance use	1	Survey	5	Health Behavior	0.3760	Illicit substance use	0.0

## Data Availability

The data presented in this study are available in publicly accessible databases, including the Research Information Sharing Service (RISS), CINAHL, MEDLINE, and PubMed. All data were derived from these public domain resources.

## Supplementary Materials

The following supporting information can be downloaded at: https://www.mdpi.com/article/10.3390/healthcare13233164/s1. Table S1. List of original and standardized keywords used in the preprocessing step. Table S2. Centrality Measures of Top Keywords in Operating Room Patient Safety Research (2020–2022). Table S3. Centrality Measures of Top Keywords in Operating Room Patient Safety Research (2023–2024).
