# Supplementary material for: Research Trends and Core Themes in Operating Room Patient Safety: A Scope-Based Keyword Network Analysis (2020–2024)
Source: Healthcare (Basel). 2025 Dec 3;13(23):3164. doi: 10.3390/healthcare13233164 (PMC12692044; doi:10.3390/healthcare13233164)
Supplement: Supplementary file 1 [file healthcare-13-03164-s001.zip › healthcare-3960288-supplementary.pdf]

Supplementary Table S1. List of original and standardized keywords used in the preprocessing step.

No	Original keyword(s)	Standardized keyword (used in analysis)	Processing note
1	Abdomen/surgery; Digestive System Surgical Procedures	Abdomen surgery	Conceptual merging
2	Adverse Health Care Event—Prevention and Control; Medical errors; Medical mistake; Patient Safety Event; Adverse Events; Adverse Event	Adverse event	Synonym unification
3	Clinical Competence; Competencies; Competency	Competency	Singularization
4	Operation Room; Surgery Room; OR; Perioperative Room	Operating room	Abbreviation expansion
5	Patient Safety; Safety of Patients	Patient safety	Terminology standardization
6	Simulation Training; Simulation-Based Learning	Simulation	Conceptual integration
7	Nurse Education; Nursing Education	Nursing education	Synonym unification
8	Infection Prevention; Infection Control	Infection control	Terminology harmonization
9	Team Collaboration; Interprofessional Cooperation; Multidisciplinary Teamwork	Teamwork	Conceptual integration
10	Artificial Intelligence; AI; Machine Learning; AI Technology	Artificial intelligence	Abbreviation expansion
11	COVID-19; Corona 19; Coronavirus disease; Covid-19	COVID-19	Terminology standardization
12	Safety Management; Safety Control	Safety management	Synonym unification

Note: Note: This table presents representative examples of the keyword preprocessing process; only major keywords are included for illustrative purposes.

Supplementary Table S2. Centrality Measures of Top Keywords in Operating Room Patient Safety Research (2020–2022)

Rank	Keyword	Degree	Betweenness	Closeness	Eigenvector	PageRank
1	Patient safety	54	0.267	0.524	1.000	0.104
2	Operating room	37	0.207	0.521	0.734	0.063
3	Perioperative care	31	0.168	0.512	0.622	0.048
4	Humans	29	0.155	0.423	0.508	0.045
5	Competency	12	0.067	0.461	0.167	0.017
6	Surgery	11	0.086	0.349	0.196	0.017
7	Anesthesia	9	0.011	0.404	0.076	0.012
8	Attitude of health personnel	9	0.017	0.374	0.177	0.012
9	Nurse	9	0.023	0.415	0.195	0.013
10	Perioperative	9	0.056	0.436	0.085	0.012
11	Risk assessment	9	0.011	0.404	0.076	0.012
12	Nursing	8	0.009	0.327	0.147	0.013
13	Safety management	8	0.014	0.454	0.106	0.010
14	Teamwork	8	0.053	0.431	0.150	0.011
15	Agitation	7	0.000	0.359	0.035	0.009
16	Atrial fibrillation	7	0.002	1.000	0.000	0.017
17	Cognition	7	0.008	0.449	0.104	0.008
18	Cross-sectional studies	7	0.008	0.449	0.104	0.008
19	Delirium	7	0.000	0.359	0.035	0.009
20	Distractions	7	0.007	0.338	0.155	0.011

Supplementary Table S3. Centrality Measures of Top Keywords in Operating Room Patient Safety Research (2023–2024)

Rank	Keyword	Degree	Betweenness	Closeness	Eigenvector	PageRank
1	Patient safety	35	0.468	0.594	1.000	0.102
2	Operating room	29	0.302	0.526	0.719	0.079
3	Humans	20	0.175	0.508	0.707	0.056
4	Operating room nurses	11	0.100	0.395	0.264	0.029
5	Health care worker	10	0.145	0.429	0.202	0.029
6	Perioperative care	10	0.068	0.324	0.374	0.031
7	Safety management	10	0.032	0.484	0.273	0.022
8	Covid-19	8	0.003	0.426	0.314	0.021
9	Health personnel	8	0.003	0.426	0.314	0.021
10	Informed consent	8	0.133	0.435	0.151	0.021
11	Nurse	8	0.043	0.472	0.179	0.019
12	Quality improvement	8	0.019	0.387	0.256	0.021
13	Organizational culture	6	0.002	0.426	0.172	0.013
14	Pandemics	6	0.002	0.426	0.172	0.013
15	Patients	6	0.002	0.313	0.276	0.018
16	Adverse event	5	0.009	0.462	0.159	0.012
17	Contamination	5	0.000	0.355	0.068	0.013
18	Nursing	5	0.000	0.355	0.068	0.013
19	Operative process	5	0.009	0.462	0.159	0.012
20	Postoperative complications	5	0.002	0.395	0.204	0.014
